# Distinct patterns of default mode network functional connectivity between adolescents with bipolar disorder and major depressive disorder

**DOI:** 10.3389/fpsyt.2026.1809961

**Published:** 2026-06-30

**Authors:** Yuxi Wang, Pengyu Zhu, Jialin Xiang, Junchen Gu, Xiong Chen, Fang Chen, Lulu Zou, Fuyi Qin, Dan Cheng, Dong Guan, Kun Qin, Chunqi Ai, Wen Chen

**Affiliations:** 1 Department of Radiology, Taihe Hospital, Hubei University of Medicine, Shiyan, China; 2 Mental Health Center, Taihe Hospital, Hubei University of Medicine, Shiyan, China

**Keywords:** adolescents, bipolar disorder, machine learning, major depressive disorder, resting-state functional magnetic resonance imaging

## Abstract

**Background:**

Bipolar disorder (BD) and major depressive disorder (MDD) exhibit overlapping clinical presentations, posing significant challenges for differential diagnosis and often leading to misidentification; therefore, elucidating the neural mechanisms that distinguish these two disorders is of critical importance.

**Methods:**

In this study, 122 adolescents (43 with BD, 39 with MDD, and 40 healthy controls) completed resting-state functional magnetic resonance imaging (rs-fMRI). Voxel-level seed-based functional connectivity (FC) analysis using default mode network (DMN) subregions and machine learning classification were applied.

**Results:**

Group-level analysis revealed that compared with healthy controls (HCs), patients with bipolar disorder (BD) exhibited significantly reduced FC between the anterior medial prefrontal cortex (aMPFC) and regions including the bilateral superior temporal gyrus (STG) and right temporal pole (TPOsup), as well as between the posterior inferior parietal lobule (pIPL) and the left middle temporal gyrus (MTG) and left STG. Relative to BD patients, patients with major depressive disorder (MDD) showed stronger FC between the aMPFC and left STG, and between the pIPL and right inferior frontal gyrus (IFG). No significant MDD–HC differences were detected in these circuits. Furthermore, no significant associations were found between altered DMN FC and clinical symptoms. Machine-learning analyses showed modest and unstable classification performance under nested 10-fold cross-validation, with a pooled out-of-fold AUC of 0.609, accuracy of 0.598, sensitivity of 0.564, and specificity of 0.628.

**Conclusion:**

Our results indicate that patients with MDD and BD exhibit distinct patterns of DMN connectivity with regions subserving sensory and cognitive processing, which may provide a potential neurobiological marker worthy of further investigation for discriminating between these disorders.

## 1 Introduction

Adolescence is a critical period of pubertal, neural, and social-emotional development, during which vulnerability to mood-related psychopathology increases, including depressive disorders and bipolar-related disorders (1, 2). Adolescent MDD, in particular, represents a major mental health concern with important neurobiological implications (3). MDD and BD share substantial clinical overlap during depressive episodes. Both conditions may present with depressed mood, loss of interest or pleasure, sleep or appetite disturbance, psychomotor agitation or retardation, fatigue, impaired concentration, feelings of worthlessness or excessive guilt, and recurrent thoughts of death or suicide. The key clinical distinction between MDD and BD lies in the presence or absence of a lifetime history of manic or hypomanic episodes. MDD is diagnosed when one or more major depressive episodes occur without any lifetime manic or hypomanic episode, whereas BD is characterized by manic episodes in BD-I or hypomanic episodes together with major depressive episodes in BD-II (4, 5). Therefore, when adolescents with BD present initially or predominantly with depressive symptoms, especially before a clear history of mania or hypomania has been identified, they may be clinically misclassified as having MDD. Such misclassification can lead to inappropriate treatment, delayed mood-stabilizing intervention, increased risk of antidepressant-induced mood switching, and poorer clinical outcomes (6–8). This clinical overlap and diagnostic uncertainty provide the foundation for investigating neural markers that may help distinguish BD from MDD during adolescence.

The DMN, an interconnected set of brain regions highly active at rest, supports internally oriented cognition, including self-referential processing and episodic simulation. However, accumulating evidence indicates substantial functional and anatomical heterogeneity within this network. Leveraging FC and task-based fMRI, Andrews-Hanna et al. proposed a parsimonious model comprising a core and two dissociable subsystems (9). This core, formed by the anterior medial prefrontal cortex (aMPFC) and posterior cingulate cortex, acts as a central hub. The subsystems display functional specialization. The dorsomedial prefrontal (dMPFC) subsystem, encompassing the dorsal medial prefrontal cortex, temporoparietal junction, and allied regions, is preferentially engaged during reflection on the present self and supports social-cognitive functions and awareness of one’s immediate mental state (10). This specialization is corroborated by intracranial neurophysiological evidence linking DMN subregions to distinct aspects of self-referential and memory processes (11). Conversely, the medial temporal lobe (MTL) subsystem, which includes the ventral medial prefrontal cortex, hippocampal formation, and connected areas, shows stronger activation during future self-prospection, highlighting its contribution to memory-based episodic simulation and prospective thought (12). The dynamic interplay between these subsystems and their coordination with other large-scale networks has been further elucidated by latent brain-state analyses of the stream of consciousness (13). Thus, the DMN supports higher-order introspection through specialized yet interacting subsystems.

The DMN is centrally implicated in self-referential thought, emotion regulation, and episodic memory processing (14). Altered DMN function is increasingly recognized as a shared pathophysiological feature across depressive spectrum disorders. In MDD, DMN abnormalities have been linked to depressed mood, altered self-representation, and pathological rumination (15, 16). Recent intracranial electrophysiological findings further suggest dysregulated engagement of specific DMN subsystems during negative self-referential processing in depression (17). Moreover, DMN FC has been proposed as a potential neuroimaging marker for differentiating BD and MDD. Prior studies indicate divergent patterns of DMN reorganization in BD and MDD, potentially reflecting distinct clinical phenotypes and underlying pathways (18). However, it remains unclear whether disorder-specific DMN connectivity signatures are already detectable during adolescence, a critical neurodevelopmental window when both disorders commonly emerge. Together, these observations motivate the search for adolescent DMN connectivity features that capture both shared and disorder-specific mechanisms.

The functional significance of the DMN is shaped not only by its intrinsic architecture but also by its interactions with other large-scale systems. DMN coupling with executive control networks involved in response inhibition and emotion regulation may provide a discriminative pathway (19). In this context, altered connectivity between posterior DMN regions and prefrontal control areas (e.g., inferior frontal regions) may contribute to disorder-specific cognitive–affective profiles, including rumination, negative self-referential processing, and impaired cognitive control. Likewise, aberrant connectivity between core DMN hubs and temporal cortices involved in social–semantic processing (e.g., superior/middle temporal regions and the temporal pole) may reflect differences in self-concept construction and interpersonal cognition across mood disorders (20, 21). During development from adolescence to adulthood, DMN FC typically matures with progressive stabilization and improved neural efficiency (22). Nevertheless, findings on DMN FC alterations in adolescents with MDD or BD remain heterogeneous, likely due to variability in age at onset, clinical course, and analytic strategies across studies. Many prior investigations have relied on a limited set of DMN seeds, typically the posterior cingulate cortex or MPFC, overlooking functional heterogeneity among DMN subsystems. Accordingly, a subsystem-informed strategy may better capture distributed DMN connectivity alterations in adolescent mood disorders. For instance, MPFC subregions support partially dissociable functions: the ventromedial prefrontal cortex contributes to affective valuation, the dorsomedial MPFC supports social cognition, and hippocampal formations provide contextual information for memory and emotional processing (23–25). Evidence from social-cognitive paradigms further supports functional specialization within DMN subsystems, particularly involving medial prefrontal regions (26).

Previous comparative neuroimaging evidence supports the relevance of DMN interactions with prefrontal control and temporal social-semantic regions in distinguishing BD from MDD. Rai et al. reported that resting-state connectivity between default-mode and fronto-parietal networks distinguished patients with BD from those with MDD, suggesting that altered coordination between internally oriented DMN processes and cognitive-control systems may be relevant to BD–MDD differentiation (19). A recent comparative meta-analysis further examined shared and distinct DMN dysconnectivity patterns in MDD and BD and demonstrated disorder-specific alterations involving frontal and temporal regions (18). These findings provide a comparative neuroimaging basis for focusing on DMN connectivity with prefrontal control regions and temporal association cortices. Functionally, the right inferior frontal gyrus is implicated in response inhibition, a key component of cognitive control (43), making it a plausible prefrontal target through which DMN–control network dyscoordination may differentiate BD from MDD. The temporal pole has been associated with language-related, semantic, autobiographical, and socio-emotional processing (37), whereas the superior temporal gyrus/sulcus region, particularly the superior temporal sulcus, is involved in social-perceptual processing, with evidence showing functional selectivity of the superior temporal sulcus for naturalistic social interaction perception (38). Therefore, altered DMN coupling with the right inferior frontal gyrus, bilateral superior temporal gyri, and temporal pole may reflect disorder-specific differences in the integration of self-referential, cognitive-control, and social-semantic processes.

Accordingly, we used a DMN subsystem-informed seed-based resting-state fMRI approach to compare whole-brain DMN functional connectivity in adolescents with bipolar disorder and major depressive disorder, with healthy controls as a reference. We hypothesized that BD–MDD differences would be most pronounced in DMN functional connectivity with prefrontal control regions, including the right inferior frontal gyrus, and with temporal cortices supporting social and semantic processing, including the bilateral superior temporal gyri and the temporal pole, differences that may aid the differential identification of BD versus MDD during adolescence.

## 2 Methods

### 2.1 Participants

A total of 90 inpatients were consecutively recruited from Taihe Hospital, China, along with 40 demographically matched healthy controls (HCs) from the local community. Of the recruited patients, 5 with MDD and 3 with BD were excluded from the analysis due to excessive head motion. Consequently, the final study sample consisted of 43 adolescents with BD, 39 with MDD, and 40 HCs. All patients were diagnosed by experienced psychiatrists using the Structured Clinical Interview for DSM-5 (SCID-5) (27). Subtypes of bipolar disorder (BD-I and BD-II) were not differentiated. Demographic information, including age, sex, education level, and illness duration, was obtained for all participants. Symptom severity was assessed using the 24-item Hamilton Depression Rating Scale (HAMD-24), the 14-item Hamilton Anxiety Rating Scale (HAMA-14), and the Young Mania Rating Scale (YMRS) (28–30). Inclusion criteria were: (1) age 12–18 years; (2) meeting DSM-5 diagnostic criteria for MDD or BD; or (3) being a healthy control matched to the patient groups. Exclusion criteria included: (1) history of significant neurological disorders (e.g., epilepsy, brain tumors) or severe systemic medical conditions; (2) history of substance abuse or current use of psychoactive substances; (3) comorbid psychiatric disorders that substantially influence brain function (e.g., severe anxiety disorders, schizophrenia); or (4) contraindications for magnetic resonance imaging (MRI). Regarding medication status, nine patients with BD (20.9%) were receiving lithium monotherapy at the time of scanning, whereas all other BD patients, all MDD patients, and all HCs were medication-free. No participant was receiving antipsychotics, antidepressants, benzodiazepines, or other psychotropic medications at the time of scanning. Precise lithium dosage, serum lithium levels, and treatment duration were not available from the clinical records. The study was approved by the Research Ethics Committee of Taihe Hospital, Shiyan, and was conducted in accordance with the Declaration of Helsinki. Written informed consent was obtained from all participants and their legal guardians.

### 2.2 MRI acquisition

The 3T GE Signa Architect scanner, including a 48-channel phased-array head coil for signal acquisition, was utilized for magnetic resonance imaging (MRI). The obtained sequences comprised high-resolution 3D T1-weighted structural imaging and fMRI. The 3D T1-weighted images were obtained using a 3D Bravo sequence with the following parameters: repetition time/echo time (TR/TE) = 2400/2.01 ms; inversion time (TI) = 1000 ms; flip angle (FA) = 8°; field of view (FOV) = 256 mm × 256 mm; matrix size = 256 × 256; slice thickness (ST) = 1 mm; voxel size = 1 mm × 1 mm × 1 mm. Resting-state functional images were acquired using a gradient-echo EPI sequence. The sequence had these parameters: TR/TE = 2000/30 ms; FA = 52°; number of axial slices = 64; ST = 3 mm, voxel size = 3 mm × 3 mm × 3 mm; FOV = 240 mm × 240 mm; matrix size = 80×80. During the resting-state fMRI scan, participants were instructed to remain awake with their eyes open and to let their minds wander without focusing on any specific thoughts.

### 2.3 Imaging data preprocessing

The fMRI data were preprocessed using Statistical Parametric Mapping 12 (SPM12) and DPABI software (31). The first ten volumes of each functional time-series were discarded to allow for magnetic field stabilization and participant adaptation to the scanning environment, leaving 230 volumes for subsequent analysis. Framewise displacement (FD) was calculated from the translational and rotational head motion parameters, as described previously (32). To rigorously control for motion artifacts, we implemented a scrubbing procedure: any volume with FD > 0.2 mm was flagged. Both the flagged volume and its immediately preceding and succeeding volumes were removed to account for the temporal spread of motion effects. A FD value of <0.2 mm for a single time point indicates good data quality. The mean FD was calculated as the average of the sum of the absolute values of the head motion estimates at each time point through backward differences. Participants were required to retain at least 160 valid time points in total after scrubbing, not necessarily consecutive volumes, for inclusion in the final analysis; those with fewer than 160 valid volumes were excluded. Subsequent preprocessing steps included slice timing correction and head motion realignment. Functional images were then normalized to the Montreal Neurological Institute (MNI) space at 3 × 3 × 3 mm³ resolution using the Diffeomorphic Anatomical Registration Through Exponentiated Lie Algebra (DARTEL) tool (33). Nuisance covariate regression was performed to remove potential confounding signals, including 24 Friston motion parameters, cerebrospinal fluid signals, white matter signals, and linear trends. Spatial smoothing was applied with a 6 mm full-width at half-maximum (FWHM) isotropic Gaussian kernel, followed by band-pass filtering (0.01–0.1 Hz) to retain low-frequency fluctuations.

### 2.4 Functional connectivity analysis

FC of the DMN was assessed using a seed-based approach. Eleven established DMN nodes were selected according to the functional-anatomic DMN parcellation reported by Andrews-Hanna et al. (9), encompassing core hubs (anterior medial prefrontal cortex, aMPFC; and posterior cingulate cortex, PCC) and two major subsystems: the dorsomedial prefrontal subsystem (dorsomedial prefrontal cortex, dMPFC; temporoparietal junction, TPJ; lateral temporal cortex, LTC; temporal pole, TempP) and the medial temporal lobe subsystem (ventromedial prefrontal cortex, vMPFC; posterior inferior parietal lobule, pIPL; retrosplenial cortex, Rsp; parahippocampal cortex, PHC; hippocampal formation, HF+). Seed coordinates are listed in **Supplementary Table 1**. Each seed was defined as a 5-mm spherical region of interest centered on the predefined MNI coordinates listed in **Supplementary Table 1**. Pearson correlation coefficients were computed between each seed’s time series and all brain voxels. Resulting r-values were converted to z-scores using Fisher’s transformation for group-level inference.

### 2.5 Machine learning analysis

**Figure 1:**
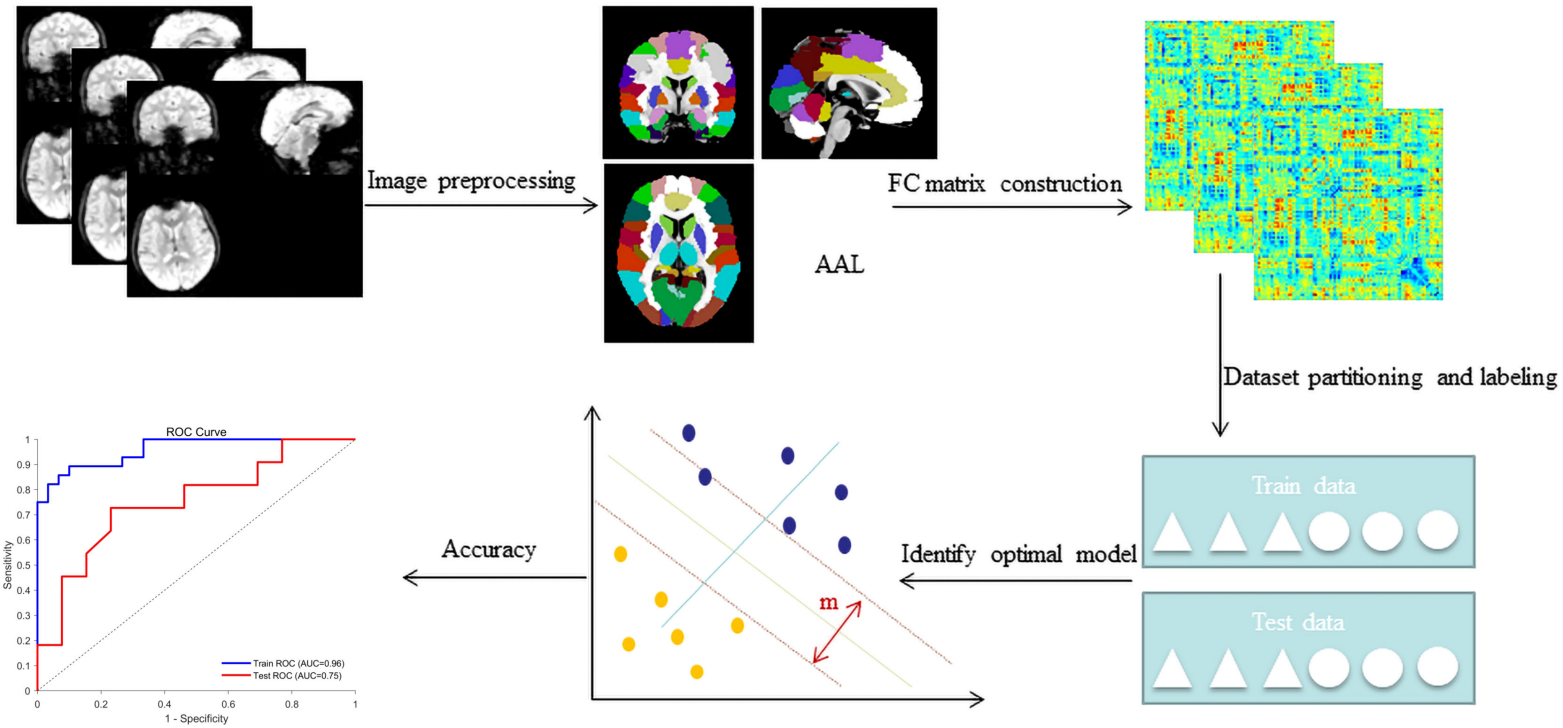
SVM classification pipeline for differentiating adolescents with MDD and BD using ROI-level DMN connectivity features. AAL, Automated Anatomical Labeling; BD, bipolar disorder; FC, functional connectivity; fMRI, functional magnetic resonance imaging; MDD, major depressive disorder; SVM, support vector machine.

A machine learning framework employing rs-fMRI was developed to discriminate patients diagnosed with MDD from those with BD. During preprocessing, FC matrices were constructed by computing Fisher’s z-transformed correlation coefficients between time series extracted from 11 predefined seed regions and 90 cerebral regions from the original Automated Anatomical Labeling atlas (AAL90) (34). Because of the modest sample size, ROI-level FC features between the 11 predefined DMN seeds and 90 AAL regions were used for machine learning classification to reduce feature dimensionality and limit overfitting risk. This ROI-level feature space was complementary to, but not identical to, the voxel-wise seed-to-voxel feature space used for group-level statistical inference. First, to retain comparability with the original analysis, the whole dataset was randomly divided into training and test subsets in a 7:3 ratio (RNG = 42). This split was performed prior to model development to ensure that the held-out test set remained independent and was not involved in feature selection, scaling parameter estimation, or model fitting. Feature selection was conducted exclusively within the training set using a LASSO-based approach with 5-fold cross-validation (35). A sparse set of features was identified, and the 10 features with the largest absolute coefficients were retained as the final feature set for subsequent classification. Classification was performed using a support vector machine (SVM) with a radial basis function (RBF) kernel. Prior to SVM training, features were rescaled to the range of 0–1 based on the training data, and the same scaling parameters were applied to the test data. The SVM hyperparameters were set to C = 10 and gamma = 0.1. Model performance was evaluated on the independent test set using accuracy, sensitivity, specificity, and the area under the receiver operating characteristic curve (AUC). MDD was defined as the positive class and BD as the negative class for the calculation of sensitivity and specificity. Sensitivity was therefore defined as the proportion of MDD patients correctly classified as MDD, whereas specificity was defined as the proportion of BD patients correctly classified as BD. Accuracy confidence intervals were calculated using exact binomial confidence intervals. The 95% confidence interval of the held-out test AUC was estimated using bootstrap resampling. To assess whether the observed test accuracy could arise under random label assignment, permutation testing with 1,000 iterations was performed by shuffling the test-set labels and recomputing accuracy while keeping the trained model fixed. This original 7:3 held-out analysis was regarded as exploratory. Second, to obtain a less biased estimate of model generalizability and to address the risk of overfitting, a nested 10-fold cross-validation procedure was performed. In each outer fold, the held-out fold was used only for final model evaluation. Within the outer training data, an inner 5-fold cross-validation was used to tune the SVM hyperparameters. The candidate values were C = 0.1, 1, 10, and 100, and gamma = 0.001, 0.01, 0.1, and 1. Feature selection, feature scaling, hyperparameter tuning, and model fitting were repeated strictly within the training data of each fold. Specifically, LASSO-based feature selection was performed only within the corresponding training fold, scaling parameters were estimated from the training fold only, and the resulting transformation was then applied to the held-out validation or test fold. This procedure was used to minimize information leakage and reduce optimistic bias in model evaluation. Nested cross-validation performance was summarized using mean outer-fold AUC, accuracy, sensitivity, and specificity, as well as pooled out-of-fold AUC, accuracy, sensitivity, and specificity. The 95% confidence interval of the pooled out-of-fold AUC was estimated using bootstrap resampling. The overall analytical workflow is schematically represented in **Figure 1**.

### 2.6 Statistical analyses

Statistical analyses for demographic and clinical data were performed using SPSS version 23.0, with the significance level set at *p* < 0.05. The Shapiro–Wilk test was used to assess normality. Consequently, comparisons among the three groups (HC, MDD, and BD) were conducted using one-way analysis of variance (ANOVA) for normally distributed data or the Kruskal–Wallis H test for non-normally distributed data, while two-group comparisons employed independent two-sample *t*-tests or Mann–Whitney U tests depending on normality. Categorical variables were analyzed using the chi-square test. For functional connectivity analysis, voxel-wise analysis of covariance (ANCOVA) was performed on the FC maps of the 11 DMN seeds using DPABI/SPM12, with age, sex, years of education, and mean FD included as covariates. To rigorously control for multiple comparisons, the significance threshold for the ANCOVA main effects was set at a voxel-level *p < 0.001* (uncorrected) and a cluster-level FWE-corrected *p* < 0.0045 (Bonferroni-corrected for 11 seeds). For clusters showing significant main effects, *post hoc* pairwise comparisons were performed within the significant mask and further thresholded at a voxel-level *p* < 0.001 (uncorrected) and a cluster-level FWE-corrected *p* < 0.016 (Bonferroni-corrected for 3 pairwise comparisons). The three *post hoc* pairwise contrasts were BD vs. MDD, BD vs. HC, and MDD vs. HC. To examine the potential influence of medication status, we conducted a sensitivity analysis excluding the nine BD patients who were receiving lithium monotherapy at the time of scanning. The whole-brain seed-to-voxel analyses for the two seeds showing significant main findings in the full sample, namely, aMPFC and pIPL, were repeated in the reduced sample using the same preprocessing pipeline, covariates, and statistical thresholds as in the main analysis.

## 3 Results

### 3.1 Demographic and clinical characteristics

As summarized in **Table 1**, the three groups (BD, MDD, and HC) were well-matched in terms of age, sex, education level, mean framewise displacement (FD), maximum FD, and the number of valid volumes retained after scrubbing, with no significant differences observed among the groups (*all p > 0.05)*. Regarding clinical features, no significant differences were found between the patient groups (MDD and BD) in symptom severity (including HAMD-24, HAMA-14, and YMRS scores), illness duration, or age of onset (*all p > 0.05*). Nine BD patients were on lithium monotherapy (**Table 1**); no participant in the MDD or HC group was receiving psychotropic medication. In the MDD group, 17 of 39 patients (43.6%) had YMRS scores ≥ 12, although none met DSM-5 criteria for bipolar disorder or a manic/hypomanic episode.

**Table 1:** Demographic and clinical characteristics of the participants.

Characteristics	BD	MDD	HC	Statistics	P-value
Case	43	39	40	N/A	N/A
Sex (M/F)	12/31	13/26	11/29	χ^2^ = 0.405^a^	0.817
Age (years old)	14.7(13.7,15.4)	14.0(14.0,15.5)	14.3(13.3,15.4)	χ^2^ = 1.567^b^	0.457
Education level (JH/SH)	33/10	29/10	33/7	χ^2^ = 0.808^a^	0.668
Age of onset (years old)	13.0(13.0,14.3)	13.5(12.4,15.0)	N/A	Z=-0.499^d^	0.618
HAMD-24 scores	32.1 ± 14.4	28.4 ± 10.1	N/A	T=1.289^c^	0.201
HAMA-14 scores	21.1 ± 9.6	22.2 ± 8.5	N/A	T=-0.528^c^	0.599
YMRS scores	13.0(10.0,19.0)	11.0(7.0,15.0)	N/A	Z=-1.749^d^	0.080
Illness duration (months)	8.0(4.0,12.0)	6.0(2.0,12.0)	N/A	Z=-1.377^d^	0.169
Mean FD (mm)	0.047(0.039,0.065)	0.051(0.042,0.074)	0.052(0.047,0.059)	χ^2^ = 1.872^b^	0.392
Max FD (mm)	0.537(0.367,0.943)	0.546(0.449,1.124)	0.706(0.388,1.275)	χ^2^ = 1.034^b^	0.596
Lithium monotherapy	9(20.9%)	0	N/A	N/A	N/A
Valid volumes after scrubbing	216(204,225)	214(202,224)	212(198,223)	χ^2^ =1.21^b^	0.547

Data are presented as mean ± standard deviation (SD) or median (25th, 75th percentiles). BD, Bipolar Disorder; MDD, Major Depressive Disorder; HC, Healthy Control; FD, Framewise Displacement; M, Male; F, Female; JH, Junior High; SH, Senior High; HAMD-24, 24-item Hamilton Depression Rating Scale; HAMA-14, 14-item Hamilton Anxiety Rating Scale; YMRS, Young Mania Rating Scale; N/A, not applicable.

^a^χ²-test. ^b^Kruskal–Wallis H test. ^c^Two-sample t-test. d: Mann–Whitney U test

### 3.2 Seed-to-voxel functional connectivity analysis

Across the 11 DMN seed-based ANCOVA analyses, only the aMPFC and pIPL seeds showed significant group main effects that survived the prespecified threshold of voxel-level *p < 0.001* (uncorrected) and cluster-level *pFWE < 0.0045*, Bonferroni-corrected for 11 seeds. For transparency and reproducibility, the seed-wise ANCOVA statistics for all 11 DMN seeds are summarized in **Supplementary Table 2**.

#### 3.2.1 Seed-based FC of the aMPFC

**Figure 2:**
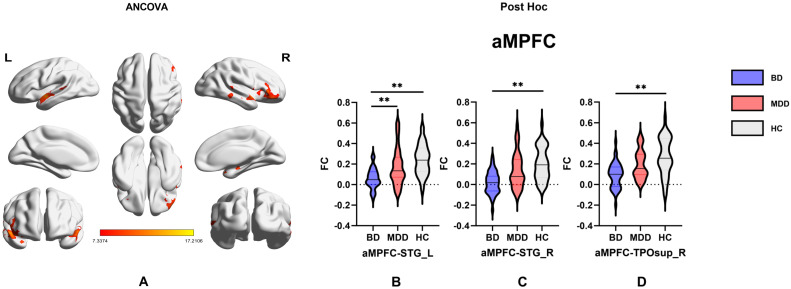
Group differences in aMPFC functional connectivity. **(A)** ANCOVA revealed significant group effects in FC between the aMPFC and the bilateral STG as well as the right TPOsup. **(B–D)** *Post hoc* analyses showed higher aMPFC–STG_L connectivity in MDD than BD, and higher connectivity in HC than BD for bilateral STG and right TPOsup. **p < 0.01. aMPFC, anterior medial prefrontal cortex; BD, bipolar disorder; HC, healthy control; MDD, major depressive disorder; STG, superior temporal gyrus; TPOsup, superior temporal pole.

The ANCOVA revealed significant group differences in the functional connectivity between the aMPFC and the bilateral superior temporal gyrus (STG) as well as the right temporal pole (TPOsup) (**Table 2**, **Figure 2**). *Post hoc* analyses indicated that the MDD group exhibited significantly increased FC with the left STG compared to the BD group. Additionally, the HC group showed higher FC with the bilateral STG and right TPOsup relative to the BD group. No significant MDD–HC differences were detected for the aMPFC seed. A small cluster in the left angular gyrus (MDD > HC) did not survive cluster-level FWE correction, and no clusters were observed for HC > MDD at the initial voxel-level threshold (*p < 0.001* uncorrected).

**Table 2:** Group differences and *post hoc* comparisons of seed-based functional connectivity.

Seed	Contrast	Brain region	Peak MNI (x y z)	Peak stat	Cluster size	Cluster-level pFWE
aMPFC	ANCOVA	STG_L	-36 -18 -9	F=17.2106	690	<0.001
aMPFC	ANCOVA	STG_R	48 3 -12	F=14.2947	947	<0.001
aMPFC	ANCOVA	TPOsup_R	69 -36 18	F=13.6812	225	<0.001
aMPFC	MDD > BD	STG_L	-42 -9 -9	T=4.4891	114	0.008
aMPFC	HC > BD	STG_L	-36 -18 -9	T=5.7062	683	<0.001
aMPFC	HC > BD	TPOsup_R	48 3 -12	T=5.2754	933	<0.001
aMPFC	HC > BD	STG_R	69 -36 18	T=5.2303	225	<0.001
aMPFC	MDD > HC	Angular_L	-45 -54 27	T=3.8757	34	0.338
pIPL	ANCOVA	MTG_L	-42 -57 -12	F=13.5741	148	0.001
pIPL	ANCOVA	STG_L	-39 -27 12	F=12.5076	345	<0.001
pIPL	ANCOVA	IFG_R	39 30 12	F=11.7633	226	<0.001
pIPL	MDD > BD	IFG_R	39 30 12	T=4.8244	128	0.007
pIPL	HC > BD	MTG_L	-42 -57 -12	T=5.0876	142	0.004
pIPL	HC > BD	STG_L	-36 -33 12	T=4.7279	324	<0.001
pIPL	MDD > HC	Cerebellum_L	-9 -72 -45	T=3.5169	12	0.870

BD, bipolar disorder; MDD, major depressive disorder; HC, healthy control; FC, functional connectivity; aMPFC, anterior medial prefrontal cortex; pIPL, posterior inferior parietal lobule; STG, superior temporal gyrus; MTG, middle temporal gyrus; IFG, inferior frontal gyrus; TPOsup, superior temporal pole; MNI, Montreal Neurological Institute; FWE, family-wise error.

#### 3.2.2 Seed-based FC of the pIPL

**Figure 3:**
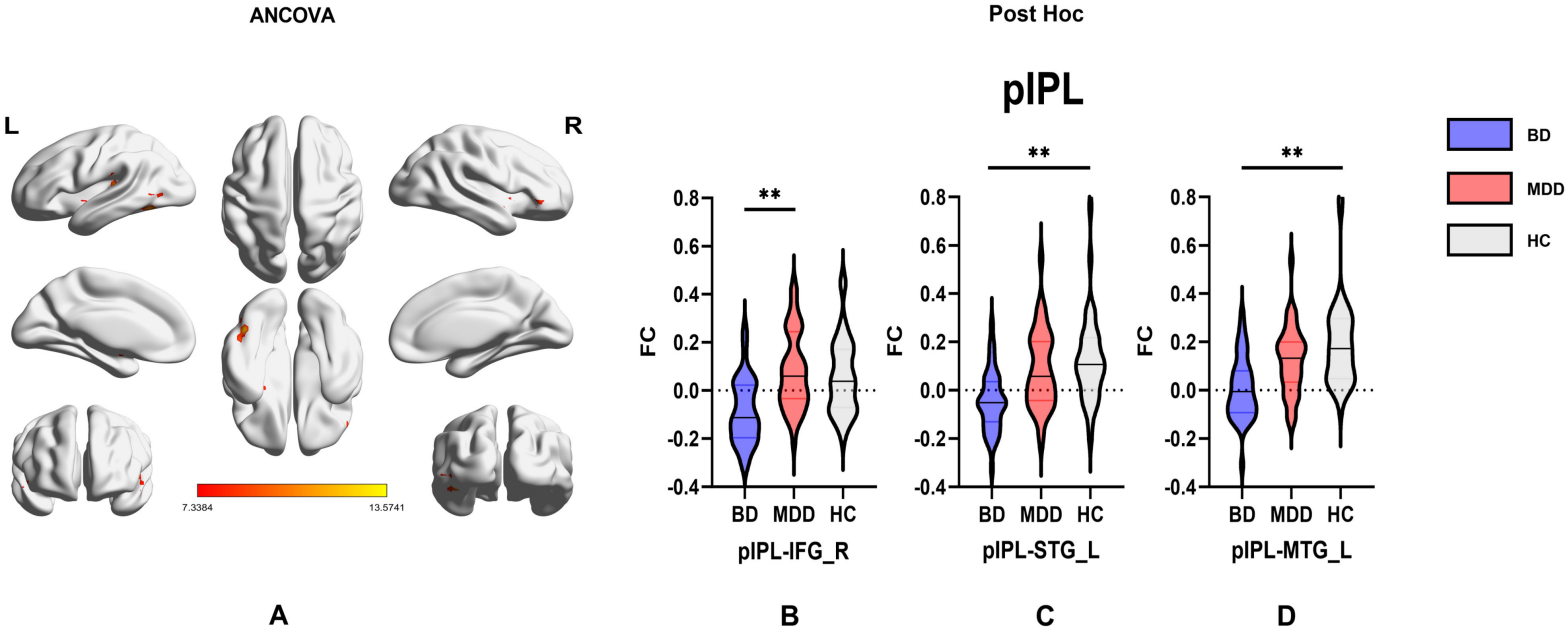
Group differences in pIPL functional connectivity. **(A)** ANCOVA revealed significant group effects in FC between the pIPL and the left MTG, left STG, and right IFG. **(B–D)** *Post-hoc* analyses showed higher pIPL–IFG_R connectivity in MDD than BD, and higher connectivity in HC than BD for pIPL–MTG_L and pIPL–STG_L. **p < 0.01. pIPL, posterior inferior parietal lobule; BD, bipolar disorder; HC, healthy control; MDD, major depressive disorder; MTG, middle temporal gyrus; STG, superior temporal gyrus; IFG, inferior frontal gyrus.

The ANCOVA results for the pIPL seed revealed significant group differences in the left middle temporal gyrus (MTG), left STG, and right inferior frontal gyrus (IFG) (**Table 2**, **Figure 3**). *Post hoc* comparisons demonstrated that the MDD group showed significantly stronger connectivity with the right IFG compared to the BD group, while the HC group exhibited increased connectivity with the left MTG and left STG relative to the BD group. No significant MDD–HC differences were detected for the pIPL seed. A small cluster in the left cerebellum (MDD > HC) did not survive cluster-level FWE correction, and no clusters were observed for HC > MDD at the initial voxel-level threshold (*p < 0.001* uncorrected).

### 3.3 Sensitivity analysis excluding lithium-treated BD participants

After excluding the nine lithium-treated BD patients, the medication-free BD subgroup consisted of 34 participants. We repeated the whole-brain seed-to-voxel analyses for the two significant seeds identified in the full-sample analysis, namely, aMPFC and pIPL, using the same covariates and statistical thresholds as in the main analysis. No clusters survived cluster-level FWE correction for either seed.

### 3.4 Association with clinical symptoms

No significant correlations were found between the altered FC values (aMPFC–STG_L and pIPL–IFG_R) and clinical ratings (HAMD-24, HAMA-14 and YMRS) in either the MDD or BD group (*all p > 0.05*).

### 3.5 Machine learning classification results

**Figure 4:**
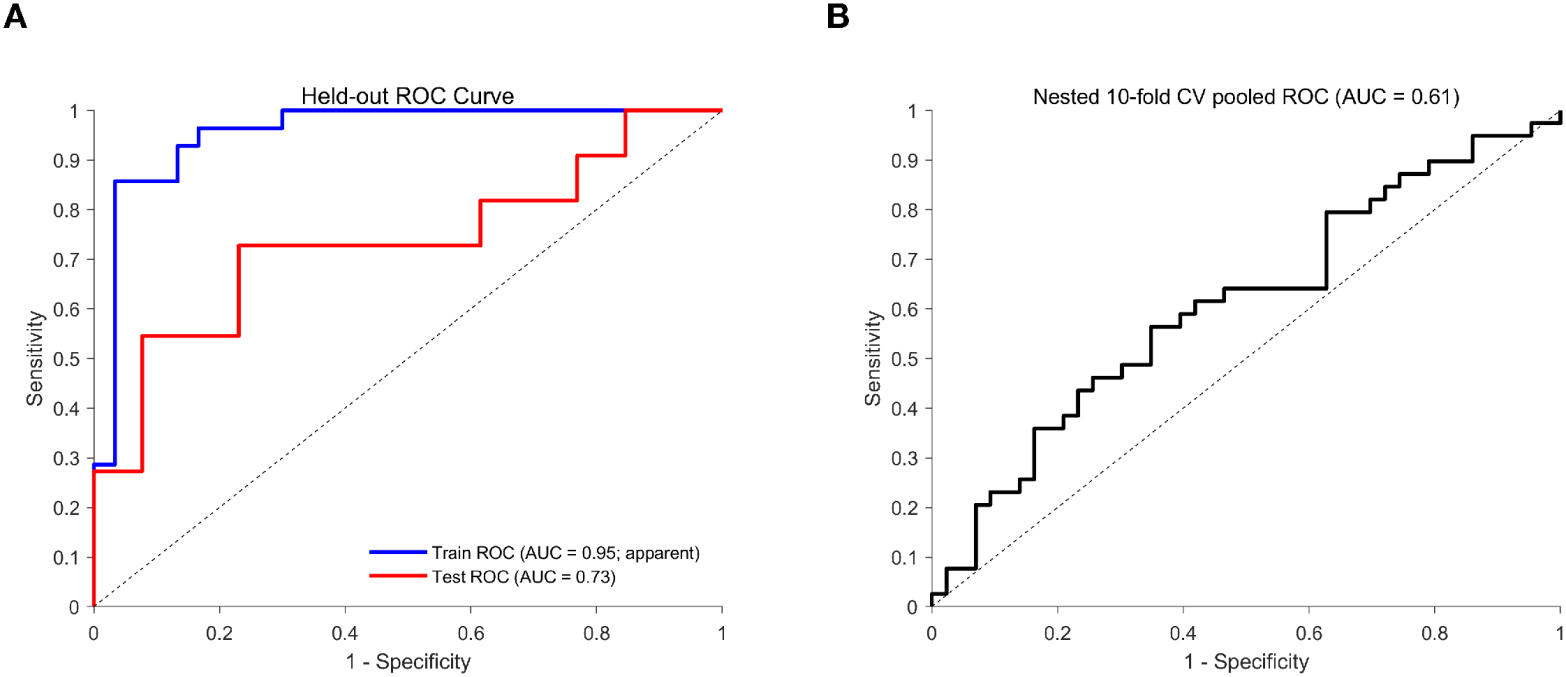
**(A)** ROC curves for the original 7:3 held-out exploratory analysis. The training ROC reflects apparent performance only, whereas the held-out test ROC reflects exploratory performance based on a small test set. **(B)** Pooled out-of-fold ROC curve from the nested 10-fold cross-validation analysis. The nested cross-validation result provides a more conservative estimate of model generalizability.

In the original 7:3 held-out exploratory analysis, the training set included 58 participants, consisting of 30 patients with BD and 28 patients with MDD, whereas the held-out test set included 24 participants, consisting of 13 patients with BD and 11 patients with MDD. MDD was defined as the positive class and BD as the negative class; therefore, sensitivity refers to the proportion of MDD patients correctly classified as MDD, and specificity refers to the proportion of BD patients correctly classified as BD. The apparent training performance was high, with an AUC of 0.955, accuracy of 0.897, sensitivity of 0.821, and specificity of 0.967. In contrast, performance on the held-out test set was lower, with an AUC of 0.734, accuracy of 0.750, sensitivity of 0.545, and specificity of 0.923 (**Figure 4A**). The bootstrap 95% confidence interval for the held-out test AUC was wide and included chance-level values, ranging from 0.491 to 0.923. The 95% binomial confidence interval for the held-out test accuracy was also wide, ranging from 0.533 to 0.902. The held-out test-set permutation test yielded p = 0.015; however, this result should be interpreted cautiously because it was based on a small held-out test set.

In the nested 10-fold cross-validation analysis, the mean outer-fold AUC was 0.581 ± 0.147, with mean accuracy of 0.596 ± 0.086, sensitivity of 0.550 ± 0.329, and specificity of 0.625 ± 0.267. The pooled out-of-fold AUC was 0.609, with a bootstrap 95% confidence interval of 0.482 to 0.725. The pooled out-of-fold accuracy, sensitivity, and specificity were 0.598, 0.564, and 0.628, respectively. The 95% binomial confidence interval for the pooled out-of-fold accuracy was 0.483 to 0.704. The discriminative ROI-level features selected in the machine learning analysis did not show exact anatomical overlap with the clusters that survived correction in the voxel-wise seed-to-voxel group analysis, although both analyses involved broadly related DMN, temporal, and frontal systems. These findings suggest that although DMN connectivity features may contain limited discriminative information, the classification performance was modest and unstable under rigorous cross-validation. Therefore, the machine-learning results should be considered exploratory and hypothesis-generating rather than evidence of established diagnostic utility.

## 4 Discussion

Our investigation integrated fine-grained DMN FC patterns using a DMN subsystem-informed seed-based rs-fMRI approach to examine functional connectivity differences among adolescents with BD, adolescents with MDD, and healthy controls. We observed distinct connectivity profiles within DMN subsystems, particularly involving the aMPFC and pIPL. Specifically, adolescents with BD exhibited marked hypoconnectivity in DMN–temporal circuits, involving reduced coupling between these DMN nodes and temporal regions (including the STG, MTG, and TPOsup) compared to HCs. In contrast, patients with MDD displayed significantly stronger FC than BD patients in specific circuits linking the aMPFC to the STG_L and the pIPL to the IFG_R. These findings suggest that adolescent BD and MDD may be associated with partially distinct patterns of DMN subsystem dysconnectivity. However, the machine-learning results should be interpreted cautiously. Although the original held-out analysis showed moderate discrimination between BD and MDD, the large discrepancy between apparent training performance and held-out test performance, together with the limited nested cross-validation performance, indicates that the classification findings are exploratory and hypothesis-generating rather than evidence of established diagnostic utility.

The aMPFC, as a core hub of the anterior DMN (9), is critically involved in self-referential processing and social appraisal (36). Its functional coupling with STG and TPOsup, regions implicated in language comprehension and social perceptual processing, including functions commonly attributed to the STG and adjacent superior temporal sulcus, is thought to support the integration of internal mental states with external social cues (37, 38). Adolescents with BD showed significantly reduced aMPFC-STG/TPOsup connectivity compared to both HCs and MDD patients. This pattern suggests a disconnection between self-focused mentation and the processing of socio-emotional signals, which aligns with clinical evidence of impaired social cognition and mentalizing deficits in pediatric BD that can persist beyond acute mood episodes (39). In contrast, MDD patients exhibited enhanced aMPFC-STG_L connectivity relative to BD, while not differing from HCs. This relatively stronger aMPFC–STG_L connectivity in MDD compared with BD may reflect a relative BD–MDD difference in the coupling between self-referential and social-semantic systems, potentially related to depressive cognitive-affective processes. Nevertheless, because the MDD group did not differ significantly from HCs in this circuit, this finding should be interpreted as a relative BD–MDD difference rather than as definitive evidence of abnormal hyperconnectivity in MDD. The formal MDD–HC comparisons revealed no clusters that survived cluster-level FWE correction. Even at the initial voxel-level threshold (*p < 0.001* uncorrected), the HC > MDD direction yielded no suprathreshold clusters. Therefore, these findings should be interpreted as differences between BD and MDD, rather than as evidence that MDD connectivity is either abnormal or definitively normal relative to HCs. The observed aMPFC–temporal connectivity pattern primarily reflects reduced connectivity in BD relative to both MDD and HCs. This pattern aligns with a recent comparative meta-analysis demonstrating that DMN hyperconnectivity with frontotemporal regions is a distinguishing feature of MDD relative to BD, driven primarily by reduced connectivity in BD (18). This dissociation underscores that BD, rather than being a more severe variant of MDD, displays a distinct pattern of anterior DMN dysconnectivity.

The pIPL findings further reinforce the dissociation between BD and MDD. As a posterior hub of the default mode network, the pIPL is critically involved in episodic memory retrieval and semantic integration, and it often interacts with left temporal regions such as the MTG and STG during autobiographical recollection and language and semantic processing (40). In the present study, adolescents with BD showed reduced pIPL connectivity with the left MTG and left STG relative to healthy controls, whereas this deficit was not observed in MDD. This pattern suggests a broader disruption in the integration of semantic and autobiographical representations in BD, which may undermine the construction and maintenance of coherent internally generated narratives and contribute to difficulties in sustaining coherent internally generated cognition (37). In contrast, the MDD group exhibited increased pIPL to right IFG functional connectivity compared with BD, while not differing from healthy controls. The right IFG is a key component of right-lateralized attention and control systems (41, 42) and is strongly implicated in inhibitory control and the regulation of prepotent responses (43). Elevated coupling between the pIPL and right IFG in MDD may therefore reflect greater recruitment of control-related mechanisms in response to internally generated distress, potentially representing a compensatory but inefficient regulatory strategy. Conversely, the absence of a similar pattern in BD may indicate a distinct impairment in coordinating default mode processes with cognitive control resources. However, because this effect was observed as a relative BD–MDD difference and not as a significant MDD–HC abnormality, its functional interpretation remains tentative. Consistent with this interpretation, the formal MDD–HC comparisons for the pIPL seed did not show any corrected significant differences, and the HC > MDD direction did not yield suprathreshold clusters at the initial voxel-level threshold.

The machine-learning findings provide only limited support for the discriminative value of DMN connectivity features in the present sample. In the original 7:3 held-out exploratory analysis, the classifier achieved moderate test-set performance; however, the held-out test set contained only 24 participants, and the bootstrapped confidence interval for the test AUC was wide. Moreover, the apparent training AUC was substantially higher than the held-out test AUC, indicating potential overfitting. When feature selection, scaling, hyperparameter tuning, and model evaluation were repeated within a nested 10-fold cross-validation framework, classification performance decreased substantially, with a pooled out-of-fold AUC of 0.609 and a 95% confidence interval that included chance-level performance. These results indicate that the original held-out performance was likely optimistic and that the present classifier has limited generalizability. Therefore, the machine-learning results should not be interpreted as demonstrating clinical diagnostic utility. Rather, they suggest that DMN connectivity patterns may contain weak discriminative information that requires confirmation in larger, independent, and preferably multisite cohorts.

Medication exposure should be considered when interpreting these findings. In the present study, nine BD patients were receiving lithium monotherapy at the time of scanning, whereas all MDD patients and HCs were medication-free. Lithium has been reported to modulate resting-state functional connectivity in BD, but its effects are circuit-dependent and cannot be simply characterized as globally increasing or decreasing connectivity (44). Existing fMRI evidence suggests that lithium may exert partially normalizing effects on prefrontal and emotion-regulation circuits (44, 45). Therefore, if lithium had a substantial effect in the present sample, it might have attenuated, rather than exaggerated, BD-related connectivity abnormalities. In this context, lithium exposure alone may not fully account for the reduced DMN-related connectivity observed in the full BD sample. However, this interpretation remains cautious because precise lithium dosage, serum lithium levels, and treatment duration were unavailable. Moreover, after excluding lithium-treated BD participants, no clusters survived whole-brain cluster-level FWE correction for either the aMPFC or pIPL seed analyses, suggesting that reduced statistical power, medication-related confounding, or both may have contributed to the findings. Thus, the present results should be regarded as preliminary and require replication in larger medication-free cohorts.

Clinically, differentiating BD from MDD during adolescence remains challenging, yet it is essential because early misclassification can delay appropriate treatment and worsen long-term functional outcomes. The present findings suggest that DMN subsystem connectivity, particularly involving aMPFC- and pIPL-related temporal and control circuits, may represent a neurobiological dimension worthy of further investigation in adolescent mood disorders. However, these connectivity patterns are not suitable for immediate clinical application. Their translational value will require validation in larger independent samples, prospective longitudinal designs, and models that link baseline connectivity profiles to subsequent diagnostic trajectories, symptom course, and functional outcomes.

Several limitations warrant consideration. First, the cross-sectional design precludes causal inferences regarding whether the observed DMN dysconnectivity represents a trait marker or a state-related effect. Longitudinal studies are needed to determine whether these connectivity patterns predict diagnostic conversion, symptom recurrence, or treatment response. In addition, the modest sample size may limit statistical power and reduce the stability of both the group-level estimates and the machine-learning results. Although the original held-out analysis suggested moderate classification performance, the small test-set size, wide confidence interval of the test AUC, large train–test AUC gap, and weak nested cross-validation performance indicate that the classifier may be overfitted and is not yet suitable for diagnostic application. In addition, 17 of 39 MDD patients (43.6%) had YMRS scores ≥ 12, suggesting possible subthreshold manic or hypomanic features in part of the MDD group. Such BD-proximal features may have made the MDD group more similar to the BD group and thus biased the observed MDD–BD differences toward the null. Second, medication exposure represents an important limitation. Nine BD patients were receiving lithium monotherapy, whereas all MDD patients and HCs were medication-free. Although the sensitivity analysis excluding lithium-treated BD participants did not yield any clusters surviving cluster-level FWE correction, this negative result may reflect reduced statistical power in the medication-free BD subgroup, medication-related confounding, or both. Therefore, residual medication-related confounding cannot be excluded. Third, BD-I and BD-II were not differentiated in the present study. This grouping was consistent with our primary aim of distinguishing adolescents with BD from those with MDD, rather than examining heterogeneity within the BD group. However, because BD-I and BD-II may differ in manic symptom severity, illness course, and neural circuit organization, the present design cannot determine whether the observed DMN connectivity alterations are shared across BD subtypes or are more strongly associated with one subtype. Future studies with larger samples and longitudinal subtype characterization are needed to examine the potential moderating effect of BD subtype. Fourth, the single-center recruitment strategy may restrict representativeness, and replication in larger, demographically diverse cohorts is required to strengthen external validity. Finally, because the present work focused on DMN-based functional coupling, future studies should examine interactions between the DMN and other large-scale networks implicated in mood regulation and cognitive control to provide a more comprehensive circuit-level account of adolescent BD and MDD.

## 5 Conclusions

This study provides preliminary evidence that adolescents with BD and MDD may differ in DMN functional connectivity patterns, particularly involving aMPFC- and pIPL-related circuits. In the full sample, BD was characterized by reduced connectivity between DMN hubs and lateral temporal regions, whereas MDD showed relatively stronger connectivity than BD in aMPFC–STG and pIPL–IFG circuits, without significant MDD–HC differences in these circuits. A machine-learning classifier using ROI-level DMN connectivity features achieved above-chance discrimination between BD and MDD on a held-out test set, suggesting the potential diagnostic relevance of DMN connectivity features. However, because the sensitivity analysis excluding lithium-treated BD participants did not reproduce significant whole-brain FWE-corrected findings, these results should be interpreted cautiously. Larger medication-free and independently replicated samples are needed before DMN connectivity can be considered a reliable neuromarker for early differential diagnosis in adolescents presenting with depressive symptoms.

## Data Availability

The raw data supporting the conclusions of this article will be made available by the authors, without undue reservation.
