# Supplementary material for: Distinct patterns of default mode network functional connectivity between adolescents with bipolar disorder and major depressive disorder
Source: Front Psychiatry. 2026 Jun 30;17:1809961. doi: 10.3389/fpsyt.2026.1809961 (PMC13364920; doi:10.3389/fpsyt.2026.1809961)
Supplement: Supplementary file 1 [file Table1.docx]

**Table S1.** **MNI coordinates of the DMN seed regions.**

| **Region Name** | **Abbreviation** | **MNI Coordinate (x, y, z, mm)** |
| --- | --- | --- |
| Anterior medial prefrontal cortex | aMPFC | -6 52 -2 |
| posterior cingulate cortex | PCC | -8 -56 26 |
| Dorsal medial prefrontal cortex | dMPFC | 0 52 26 |
| temporal parietal junction | TPJ | -54 -54 28 |
| Lateral temporal cortex | LTC | -60 -24 -18 |
| Temporal pole | TempP | -50 14 -40 |
| Ventral medial prefrontal cortex | vMPFC | 0 26 -18 |
| posterior inferior parietal lobule | pIPL | -44 -74 32 |
| Retrosplenial cortex | Rsp | -14 -52 8 |
| Parahippocampal cortex | PHC | -28 -40 -12 |
| Hippocampal formation | HF+ | -22 -20 -26 |
