# Supplementary material for: Distinct patterns of default mode network functional connectivity between adolescents with bipolar disorder and major depressive disorder
Source: Front Psychiatry. 2026 Jun 30;17:1809961. doi: 10.3389/fpsyt.2026.1809961 (PMC13364920; doi:10.3389/fpsyt.2026.1809961)
Supplement: Supplementary file 2 [file Table2.docx]

**Table S2. Summary of seed-wise ANCOVA results across all 11 DMN seeds**

| **Seed** | **Contrast** | **Brain region** | **Peak MNI (x y z)** | | **Peak stat** | **Cluster size** | **Cluster-level P_FWE_** |
| --- | --- | --- | --- | --- | --- | --- | --- |
| aMPFC | ANCOVA | STG_L | | -36 -18 -9 | F=17.2106 | 690 | ＜0.001 |
| aMPFC | ANCOVA | STG_R | | 48 3 -12 | F=14.2947 | 947 | ＜0.001 |
| aMPFC | ANCOVA | TPOsup_R | | 69 -36 18 | F=13.6812 | 225 | ＜0.001 |
| PCC | ANCOVA | Heschl_L | | -36 -27 9 | F=16.0375 | 87 | 0.014 |
| dMPFC | ANCOVA | IFG_L | | -39 24 12 | F=17.1515 | 52 | 0.098 |
| TPJ | ANCOVA | IFG_L | | -45 30 9 | F=19.0276 | 36 | 0.266 |
| LTC | ANCOVA | ITG_L | | -48 -27 -24 | F=23.5500 | 35 | 0.251 |
| TempP | ANCOVA | MFG_L | | -42 45 18 | F=16.1243 | 58 | 0.064 |
| vMPFC | ANCOVA | IFG_L | | -21 24 -15 | F=23.1950 | 112 | 0.005 |
| pIPL | ANCOVA | MTG_L | | -42 -57 -12 | F=13.5741 | 148 | 0.001 |
| pIPL | ANCOVA | STG_L | | -39 -27 12 | F=12.5076 | 345 | ＜0.001 |
| pIPL | ANCOVA | IFG_R | | 39 30 12 | F=11.7633 | 226 | ＜0.001 |
| Rsp | ANCOVA | STG_R | | 54 -33 3 | F=18.5231 | 111 | 0.007 |
| PHC | ANCOVA | CAL_R | | 27 -66 6 | F=18.5334 | 76 | 0.036 |
| HF^+^ | ANCOVA | STG_L | | -39 -3 -15 | F=21.4016 | 112 | 0.007 |

**Note: ANCOVA was performed separately for each of the 11 DMN seeds with age, sex, years of education, and mean framewise displacement included as covariates. Cluster-level PFWE values are reported for each cluster. According to the prespecified correction threshold of cluster-level PFWE < 0.0045, Bonferroni-corrected for 11 seeds, only clusters associated with the aMPFC and pIPL seeds survived correction across seeds.**
